# Cognitive impairment in the population-based ural very old study

**DOI:** 10.3389/fnagi.2022.912755

**Published:** 2022-07-19

**Authors:** Mukharram M. Bikbov, Gyulli M. Kazakbaeva, Ellina M. Iakupova, Songhomitra Panda-Jonas, Albina A. Fakhretdinova, Azaliia M. Tuliakova, Iuliia A. Rusakova, Jost B. Jonas

**Affiliations:** ^1^Ufa Eye Research Institute, Ufa, Russia; ^2^Ural Ophthalmology Institute, Ufa, Russia; ^3^Privatpraxis Prof. Jonas und Dr. Panda-Jonas, Heidelberg, Germany; ^4^Department of Ophthalmology, Medical Faculty Mannheim, Heidelberg University, Mannheim, Germany; ^5^Institute of Molecular and Clinical Ophthalmology Basel, Basel, Switzerland

**Keywords:** cognitive impairment, dementia, Alzheimer’s disease, Mini-Mental State Examination, population-based study, Ural Very Old Study, epidemiology

## Abstract

**Background:**

Despite its marked importance in public health, the prevalence of cognitive impairment (CI) and its associated factors have only rarely been examined in old populations in general or in Russia at all.

**Objective:**

To assess CI prevalence and its determinants in a very elderly population in Russia.

**Materials and methods:**

The population-based Ural Very Old Study, conducted in rural and urban region in Bashkortostan/Russia, included 1,526 (81.1%) out of 1,882 eligible individuals aged 85+ years. A series of medical examinations including the Mini-Mental State Examination (MMSE) for the assessment of CI was performed.

**Results:**

Mini-Mental State Examination data were available for 1,442 (94.5%) individuals (mean age: 88.3 ± 2.9 years; range: 85–103 years). The median MMSE score was 24 (interquartile range: 19, 27). Prevalence of any CI (MMSE score < 24 points) was 701/1,442 [48.6%; 95% confidence interval (CI): 46.0, 51.2]. Prevalence of mild, moderate and severe CI (MMSE score 19–23 points, 10–18 points, and ≤9 points, respectively) was 357/1,442 (24.8%; 95% CI: 22.5, 27.0), 246/1,442 (17.1%; 95% CI: 15.1, 19.0), and 98/1,442 (6.8%; 95% CI: 5.5, 8.1), resp. A lower MMSE score correlated (regression coefficient r^2^: 0.31) with older age (beta: −0.13; *P* < 0.001), rural region of habitation (beta: 0.15; *P* < 0.001), lower level of education (beta: 0.19; *P* < 0.001), higher depression score (beta: −0.33; *P* < 0.001) (or alternatively, higher prevalence of hearing loss (beta: −0.10; *P* = 0.001), worse visual acuity (beta: −0.10; *P* = 0.001), and lower physical activity (beta: 0.06; *P* = 0.04).

**Conclusion:**

In this elderly study population from rural and urban Russia, prevalence of any, mild, moderate and severe CI was 48.6, 24.8, 17.1, and 6.8%, resp. Besides medical and lifestyle factors, vision and hearing impairment were major factors associated with CI.

## Introduction

Due to aging and simultaneous growth of the world population, cognitive impairment (CI) including Alzheimer’s disease has an increasing importance in public health, and the potential role of its modifiable risk factors has increasingly been explored in recent investigations (Gbd, 2016a; Wu et al., 2017; Gbd 2017 Us Neurological Disorders Collaborators et al., 2021). In the Global Burden of Disease Study 2019, Alzheimer’s disease and other dementias, after ischemic heart disease, stroke and chronic obstructive pulmonary disease, were the fourth most common cause of global disability-adjusted life-years (DALYs) and caused 5.6% of all DALYs (Gbd, 2019). The 2020 update of the Lancet commission on dementia prevention, intervention and care listed low education, hypertension, hearing impairment, smoking, obesity, depression, physical inactivity, diabetes, social isolation, excessive alcohol consumption, head injury, and air pollution as modifiable risk factors for dementia (Livingston et al., 2020). Knowing the prevalence of CI in the population to estimate the importance of the issue and addressing modifiable factors through public health interventions is a way to reduce the impact of CI for the individual and for the public.

Although Eastern Europe, Central Asia, and Russia form a major world region, studies examining the prevalence of CI including dementia have only scarcely been conducted, and globally most studies on the prevalence of CI and its associated factors were focused on individuals usually younger than 85 years (Gbd, 2016b; Turusheva et al., 2017). To address this lack in knowledge, we performed a population-based study in Russia on the prevalence and associated factors of CI and included individuals with a minimal age of 85 years. Goals of the study were to examine the prevalence of CI in the elderly population in Russia and to explore associations of CI with medical, socioeconomic and other parameters, taking into account interdependencies between these variables.

## Materials and methods

The Ural Very Old Study (UVOS) is a population-based study which was conducted in the Russian republic of Bashkortostan about 1,400 km East of Moscow in the period from November 2017 to December 2020 (Bikbov et al., 2021a,b). Study areas were the urban district of Kirovskyi in Ufa, the capital of Bashkortostan, and the rural region of the Karmaskalinsky District in a distance of 65 km from Ufa. Inclusion criteria were an age of 85+ years and living in the study regions, while there were no exclusion criteria. We chose a minimum age of 85 years as inclusion criterion, since most previous studies had included only few individuals with an age of 85+ years. Individuals living in retirement homes (there were three small private retirement homes in the urban study region and no retirement homes in the rural study region) were fully eligible. The Ethics Committee of the Academic Council of the Ufa Eye Research Institute approved the study and informed written consent was obtained from all participants. If individuals could not fully understand or were not fully informed about the meaning of the survey and the examination procedure applied in the study, family members or study personal repeatedly explained it to them in separate sessions until the participants were aware of the study. If the individuals were cognitively markedly impaired, informed consent was additionally obtained from family members.

### Participant recruitment

The participation in the study was supported by the local authorities. The President of the Republic of Bashkortostan asked the chairman of the Council of Ministers, the Minister of Health and the Academy of Sciences of the Republic of Bashkortostan to provide methodological and financial assistance in conducting the study. Additionally, the head of the city of Ufa and the heads of the Kirovsky district of Ufa and the rural Karmaskalinsky district of the Republic of Bashkortostan were instructed to provide all possible assistance for conducting the survey. A group of altogether 25 scientists, medical doctors and nurses became directly involved in conducting the survey. The head of the city of Ufa, the head of the Kirovsky district of Ufa and the head of the rural Karmaskalinsky district gave instructions to inform the whole population of the study regions about the survey. The administrations of the districts provided a list of the residents aged 85+ years and living in the study regions. In the Kirovsky district of Ufa, the employees of the Polyclinic No. 52 - district doctors and district nurses - were involved in informing the population about the survey and an apartment-by-apartment approach was conducted to encourage the individuals participating in the study. Also, the social services of the district, housing and communal services and other public services including the postal service took part in informing the population. Informational posters about the population survey were placed in shops and public areas. In the rural Karmaskalinsky district, the employees of the Karmaskalinsky Central District Hospital, paramedics of village paramedic stations, employees of the postal service, district police officers, rural school teachers and social workers informed the population about the survey. The administration of the Karmaskalinsky district allocated buses for the daily transport of the study participants from their place of residence to the Ufa Eye Research Institute and back. Free lunch was provided in the Ufa Eye Research Institute. Individuals who could not travel to the Ufa Eye Research Institute from the rural Karmaskalinsky district were examined in the paramedic stations, where all the necessary diagnostic equipment was delivered from the Ufa Eye Research Institute. Those individuals who were too immobile to travel to the paramedic station, were examined at their homes using mobile diagnostic equipment. All respondents who participated in the study were permanent residents. Participation in the study was voluntary, without any disadvantages for those individuals who did take part in the study. Temporary residents were not included in the study. To attract remaining individuals in the last stage of the survey who had not participated in the study yet, despite having been contacted several times by visits to their apartments, incentives were used in the form of issuing bonus cards, gifts, or certificates.

### Study participants

Out of 1,882 individuals fulfilling the inclusion criteria, 1,526 (81%) persons took part in the study. The participation rate did not vary significantly (*P* = 0.65) between the urban study region [1,238 (81.3%) out of 1,523 individuals] and the rural study region [288 (80.2%) out of 359 individuals] (Bikbov et al., 2021a,b). According to the latest census carried out in Russia in 2010, the demographic structure of the study population with respect to gender and age corresponded to the gender and age distribution in the Russian population beyond an age of 85+ years, with a marked preponderance of females (Federal State Statistic Service, 2010).

### Questionnaire

Ophthalmologists conducted an interview containing about 300 questions (Zung, 1965; Folstein et al., 1975; Radloff, 1977; Fries et al., 1982; Crum et al., 1993; Klein et al., 2001; Aiello et al., 2011; Creavin et al., 2016; Bikbov et al., 2019b; Arevalo-Rodriguez et al., 2021). Cognitive function was assessed applying Folstein’s Mini-Mental State Examination (MMSE) with a maximal score of 30 points (Folstein et al., 1975; Crum et al., 1993; Creavin et al., 2016; Arevalo-Rodriguez et al., 2021). Any score of ≥24 points indicated a normal cognition, while mild cognitive impairment, moderate cognitive impairment and severe cognitive impairment were defined by a score of 19–23 points, 10–18 points, and ≤9 points, respectively (Crum et al., 1993; Creavin et al., 2016; Arevalo-Rodriguez et al., 2021). The study participants, in particular those with hearing or vision impairment, were particularly assisted in addressing the tasks of the MMSE. For participants with a low binocular best corrected visual acuity, markedly enlarged drawings were used for the test questions.

The interview further included questions on the socioeconomic background including level of education (categorized into illiteracy, having passed the 5th, 7th, 10th, and 11th class, specialized secondary education, graduation, and post graduation), diet, smoking, alcohol consumption, physical activity, quality of life and quality of vision, symptoms of chronic obstructive pulmonary disease, asthma, kidney disease and orthopedic disorders, history of any type of injuries and inter-personal violence, health assessment questions, and history of major medical disorders. The questions had been validated in previous investigations such as the National Eye Institute Visual Functioning Questionnaire-25 (VFQ-25) (Table 1 and Supplementary Table 1; Klein et al., 2001). Depression was assessed applying the Center for Epidemiologic Studies Depression Scale Scoresheet (Radloff, 1977). It consisted of 20 questions including questions such as “I was bothered by things that usually do not bother me” and “I could not get going”. The participants underwent the test with the full choice of answers ranging between “0” for “rarely or none of the time (less than 1 day)”, “1” for “some or a little of the time (1–2 days), “2” for “occasionally or a moderate amount of time (3–4 days)”, and “3” for “most or all of the time (5–7 days)”. The maximum score was 60. Hearing loss was examined by a series of 11 standardized questions, 10 of which were derived from the “Hearing Handicap Inventory for the Elderly Screening Version (HHIE-S)” (Aiello et al., 2011; Bikbov et al., 2019b). Physical activity was assessed asking questions such “In your leisure time, do you do any physically vigorous activities like running, strenuous sports or weight lifting for at least 10 mins at a time?” and “In your leisure time, do you do any moderate intensity activities like brisk walking, cycling, or swimming for at least 10 mins at a time?”. We applied the Stanford Health Assessment Questionnaire (HAQ) for examining the general health of the study participants (Fries et al., 1982). The eight categories assessed by the Disability Index are (1) dressing and grooming, (2) arising, (3) eating, (4) walking, (5) hygiene, (6) reach, (7) grip, and (8) common daily activities. For each of these categories, patients reported the amount of difficulty they had in performing two or three specific activities. All questions of the questionnaire had been translated into Russian language, and the whole communication with the study participants was performed in Russian or Bashkir language. All study participants were fluent in Russian. In the situation that study participants could not immediately fully understand the questions for any reason, the interviewers took additional time to fully explain the question to the participants, or family members helped the study participants to respond to the questions.

**TABLE 1:** Demographic, lifestyle-related, diet, physical activity-related, health assessment-related, and other parameters (mean ± standard deviation or numbers and percentage in brackets) in the Ural Very Old Study, stratified between the rural part and the urban part.

	Total	Rural region	Urban region	*P*-value
*n*	1,442	266	1,176	
Age (years)	88.3 ± 2.9	88.3 ± 3.0	88.4 ± 2.9	0.42
Gender (men/women)	373 (25.9%)/1,069 (74.1%)	63 (23.7%)/203 (76.3%)	310 26.4%)/866 (73.6%)	0.39
Ethnicity (Bashkir/Russian/Tatar/Chuvash/ Mari/Other)	161 11.2%)/524 (36.3%)/646 (44.8%)/43 (3.0%)/6 (0.4%)/57 (4.0%)	50 (18.8%)/35 (13.2%)/145 (54.5%)/33 (1.4%)/1 (0.4%)/1 (0.4%)	111 (9.4%)/489 (41.6%)/501 (42.6%)/10 (0.9%)/5 (0.4%)/56 (4.8%)	
Ethnicity (Non-Russian/Russian)	913 63.3%)/524 (36.3%)	230 (86.5%)/35 (13.2%)	683 (58.1%)/489 (41.6%)	<0.001
Body height (cm)	157.4 ± 9.2	160.4 ± 8.5	156.4 ± 9.2	<0.001
Body weight (kg)	65.8 ± 11.4	64.3 ± 10.5	66.4 ± 11.7	0.01
Body mass index (kg/m^2^)	26.6 ± 4.5	25.0 ± 3.9	27.1 ± 5.6	<0.001
Waist circumference (cm)	92.1 ± 11.5	85.5 ± 12.9	94,1 ± 102	<0.001
Hip circumference (cm)	98.4 ± 10.6	93.0 ± 10.5	100.1 ± 10.0	<0.001
Waist/hip circumference ratio	0.94 ± 0.08	0.92 ± 0.10	0.94 ± 0.08	0.01
Level of education (illiteracy (no reading ability at all)/passing of the 5th class/passing of the 8th class/passing of the 10th class/passing of the 11th class/graduation/post-graduation	39 (2.7%)/276 (19.1%)/322 (22.3%)/69 (4.8%)/37 (2.6%)/313 (21.7%)/373 (25.9%)/4 (0.3%)	22 (8.3%)/111 (41.7%)/83 (31.2%)/7 (2.6%)/1 (0.4%)/24 (9.0%)/13 (4.9%)/0 (0%)	17 (1.4%)/165 (14.0%)/239 (20.3%)/62 (5.3%)/36 (3.1%)/289 (30.6%)/360 (30.6%)/4 (0.3%)	<0.001
Smoking, currently (no/yes)	1,429 (99.1%)/11 (0.8%)	262 (98.5%)/3 (1.1%)	1,167 (99.2%)/8 (0.7%)	0.44
Alcohol consumption, any (no/yes)	1,275 (88.4%)/166 (11.5%)	246 (92.5%)/20 (7.5%)	1,029 (87.5%)/146 (12.4%)	0.03
Serum concentration of high-density lipoproteins (mmol/L)	1.74 ± 0.79	1.16 ± 0.49	1.91 ± 0.78	<0.001
Serum concentration of low-density lipoproteins (mmol/L)	2.96 ± 1.08	3.41 ± 1.04	2.83 ± 1.06	<0.001
Serum concentration of cholesterol (mmol/L)	5.64 ± 1.28	5.23 ± 1.22	5.76 ± 1.28	<0.001
Prevalence of diabetes mellitus (fasting blood glucose concentration of ≥7.0 mmol/L or self-reported history of physician diagnosis of diabetes mellitus or history of drug treatment of diabetes)	907 62.9%)/190 (13.2%)	204 (76.7%)/39 (14.7%)	703 (59.8%)/151 (12.8%)	0.63
Anemia (serum hemoglobin concentration < 140 g/L in men, <130 g/L in women)	467 (32.4%)/583 (40.4%)	96 (36.1%)/148 (55.6%)	371 (31.5%)/435 (37.0%)	0.07
Blood pressure, systolic (mm Hg)	156.0 ± 26.0	163.0 ± 29.5	153.9 ± 24.5	<0.001
Blood pressure, diastolic (mm Hg)	79.3 ± 14.0	83.9 ± 15.0	77.9 ± 13.4	<0.001
Blood pressure, mean (mm Hg)	104.9 ± 15.9	110.2 ± 17.7	103.2 ± 15.0	<0.001
Arterial hypertension (no/yes)	146 (10.1%)/931 (64.6%)	33 (12.4%)/222 (83.5%)	113 9.6%)/709 (60.3%)	0.83
Arterial hypertension, stages (normal blood pressure (BP) (systolic BP (SBP) < 120 mm Hg, diastolic BP (DBP) < 80 mm Hg)/elevated BP (SBP: 120–129 mm Hg, DBP < 80 mm Hg)/stage 1 hypertension (SBP: 130–139 mm Hg, DBP: 80–89 mm Hg)/stage 2 hypertension (SBP ≥ 140 mm Hg or DBP ≥ 90 mm Hg)	68 (4.7%)/78 (5.4%)/138 (9.6%)/609 (42.2%)/184 (12.8%)	17 (6.4%)/16 (6.0%)/23 (8.6%)/125 (47.0%)/74 (27.8%)	51 (4.3%)/62 (5.3%)/115 (9.8%)/484 (41.2%)/110 (9.4%)	<0.001
Hearing loss score	19.2 ± 15.4	24.8 ± 14.4	17.9 ± 15.3	<0.001
Depression Score	7.1 ± 9.9	8.9 ± 9.4	6.7 ± 9.4	<0.001
State-Trait Anxiety Inventory	1.3 ± 10.3	2.6 ± 9.8	1.0 ± 10.4	0.002
Manual dynamometry, right hand	13.8 ± 7.5	11.3 ± 6.9	14.6 ± 7.5	<0.001
Manual dynamometry, left hand	10.7 ± 6.9	8.3 ± 6.6	11.4 ± 6.8	<0.001
Best corrected visual acuity (logarithm of the minimal angle of resolution)	0.58 ± 0.69	0.83 ± 1.05	0.49 ± 0.51	<0.001

Both groups were compared applying Student’s t-test for unpaired samples or the Wilcoxon-Mann-Whitney test.

### Examinations

As also described in detail previously, the physical and medical examinations consisted of the measurements of anthropomorphic parameters such as body height and weight and waist and hip circumference, arterial blood pressure and pulse rate, dynamometric assessment of the handgrip strength, and measurement of presenting and best corrected visual acuity (Bikbov et al., 2021a,b). Blood samples taken under fasting conditions were biochemically examined. Arterial hypertension was defined according to the guidelines of the American College of Cardiology/American Heart Association, and diabetes mellitus was characterized by a fasting blood glucose concentration of ≥7.0 mmol/L or self-reported history of physician diagnosis of diabetes mellitus or history of drug treatment of diabetes (Whelton et al., 2018). We applied the Guidelines for Accurate and Transparent Health Estimates Reporting (GATHER statement guidelines) (Stevens et al., 2016). The UVOS design was similar to the design of the Ural Eye and Medical Study (UEMS), which has been described in detail previously (Bikbov et al., 2019a,^2020^).

### Statistical analysis

Using a statistical software package (SPSS for Windows, version 27.0, SPSS, Chicago, IL, United States), we determined the median value of the MMSE score and its interquartile range (IQR) and the mean prevalence of the various degrees of cognitive impairment [presented as mean and 95% confidence intervals (CIs)]. The required size of the study population was predetermined by the selection of representative study regions and an assumed rate of participation of at least 80%. Based on the experience of previous population-based investigations on the prevalence and associated factors of major diseases (including diabetes mellitus, arterial hypertension, neurological, and ophthalmological disorders) and considering the inclusion criterion in the present study of an age group of 85+ years, we arrived at a required study sample size of 1,400+ individuals. We performed univariate analyses of the relationships between the MMSE score or the cognitive impairment grades with all other parameters measured in the study. It was followed by multivariable regression analyses with the MMSE score as the dependent parameter (linear regression analyses) or the prevalence of cognitive impairment as the dependent variables (binary regression analysis), and as independent variables all those parameters that were significantly (*P* < 0.05) correlated with the dependent variable in the univariate analyses. Out of the list of independent variables, we then dropped, in a step-by-step manner, all those parameters, which either showed a high collinearity (as expressed by the variance inflation factor) or which were no longer significantly associated with the dependent parameter. We then added various single parameters again to the list of independent variables to re-test their association with the dependent variable. We additionally performed a multinomial logistic model to assess associations between the prevalence of the various CI stages and other parameters. We calculated the standardized regression coefficient beta, the non-standardized regression coefficient B and its 95% CI, and odds ratios (ORs) and their 95% CIs. All *P*-values were two-sided and considered statistically significant when the values were less than 0.05.

## Results

Out of 1,526 individuals primarily participating in the UVOS, the present investigation included 1,442 (94.5%) individuals [1,069 (74.1%) women; 373 (25.9%) men] who underwent a complete MMSE. All study participants, as also the whole population of the republic of Bashkortostan, were fluent in Russian, and all study examiners were fluent in Russian and Bashkir language. The individuals with complete MMSE data (as compared with those without complete MMSE examination (n = 84) had a significantly higher level of education (*P* = 0.003), while they did not differ significantly in age (*P* = 0.32) and sex (*P* = 0.30). The reason for 84 individuals not having a complete MMSE were binocular severe vision impairment or blindness (*n* = 80) or illiteracy (*n* = 12) (with 8 out of the 80 individuals with binocular severe vision impairment or blindness being additionally illiterate). Due to their disability, they could not address the task “making up and writing a sentence about anything” and the task of copying a picture. While all individuals (*n* = 1,442) visited in their homes underwent the MMSE, the medical examinations and laboratory testing were performed for those individuals (*n* = 1,051; 72.9%) who could come to the hospital for these examinations. This group was younger than the group of individuals who could not come to the hospital (88.2 ± 2.8 versus 88.6 ± 3.1 years; *P* = 0.04).

The median MMSE score was 24 (IQR: 19, 27) (range: 0–30; Figure 1). A higher MMSE correlated with a lower Health Assessment Questionnaire score (Figure 2). The MMSE was significantly lower in the subgroup of participants (*n* = 107) reporting about marked memory difficulties than in the subgroup of individuals (*n* = 1,335) without self-reported memory problems (11.7 ± 9.2 versus 22.7 ± 5.8; *P* < 0.001). Correspondingly, the Health Assessment Questionnaire score was higher in the subgroup of participants with marked self-reported memory problems (16.1 ± 6.7 versus 9.6 ± 5.8; *P* < 0.001). A MMSE score of less than 24 points was achieved by 701 participants (48.6%; 95% CI: 46.0, 51.2), a score between 23 points and 19 points by 357 participants (24.8%; 95% CI: 22.5, 27.0), a score between 18 and 10 points by 246 individuals (17.1%; 95% CI: 15.1, 19.0), and a score of less than 10 points by 98 participants (6.8%; 95% CI: 5.5, 8.1). Out of the 1,442 study participants, 107 (7.4%; 95% CI: 6.1, 8.8) indicated in the interview that they had considerable problems in remembering recent events indicating some degree of cognitive dysfunction. Asked about their worst experience in their life, 55.6% reported about bad experiences during the Second World war or shortly after it, 30.3% health problems, 10.5% mentioned family problems, and 2.6% mentioned other problems. In univariable analysis, a lower cognitive score was associated with numerous parameters, such as older age (*P* < 0.001), rural region of habitation (*P* < 0.001), female sex (*P* = 0.02), higher hearing loss score (*P* < 0.001), higher depression score (*P* < 0.001), higher Strait-Trait Anxiety Inventory score (*P* < 0.001), lower hand grip force (*P* < 0.001), and worse visual acuity (*P* < 0.001; Supplementary Table 1). Those participants with a positive history of cognitive dysfunction had a significantly lower cognitive score than the other study participants [11.7 ± 9.2 (median: 11) versus 22.7 ± 5.8 (median: 24)].

**FIGURE 1:**
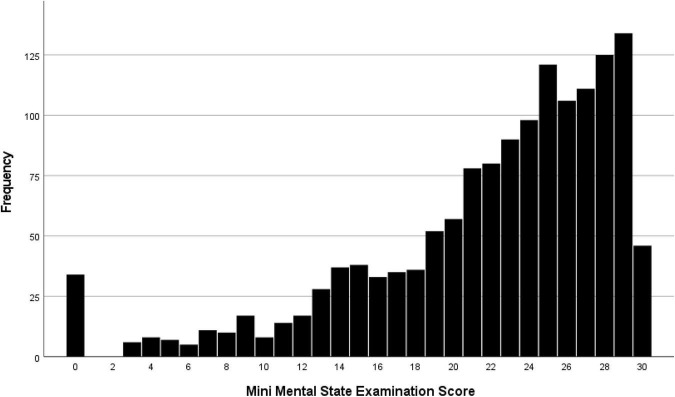
Distribution of the Mini-Mental State Examination score in the Ural Very Old Study.

**FIGURE 2:**
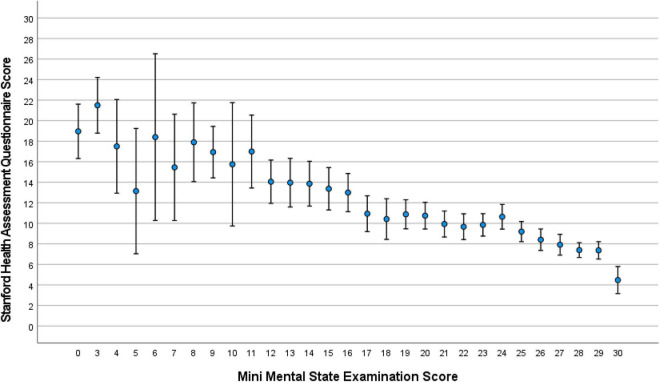
Distribution of the Stanford Health Assessment Questionnaire score, stratified by the Mini-Mental State Examination score, in the Ural Very Old Study.

In multivariable analysis, we first dropped due to collinearity the parameters of anxiety score [variance inflation factor (VIF): 4.2], and systolic and diastolic blood pressure (VIF: 3), before we dropped additional parameters due to a lack of statistical significance. In the final model, a lower MMSE score was related (regression coefficient *r*^2^: 0.31) with older age (*P* < 0.001), rural region of habitation (*P* < 0.001), lower level of education (*P* < 0.001), higher depression score (*P* < 0.001), worse visual acuity (*P* = 0.001), lower physical activity (*P* = 0.04), lower systolic blood pressure (*P* < 0.001), and higher diastolic blood pressure (*P* = 0.03; Table 2). Most of the associations, except the relationship with lower physical activity, remained to be statistically significant if a Bonferroni correction for performing multiple statistical comparisons was carried out. If the parameters of sex (*P* = 0.65), body mass index (*P* = 0.13), diabetes (*P* = 0.60), serum glucose concentration (*P* = 0.61), current smoking (*P* = 0.82), current alcohol consumption (*P* = 0.47), and family structure (*P* = 0.34), were separately added to the model, these parameters were not significantly associated with the MMSE score. If the parameter of depression was dropped, a lower MMSE was additionally associated with a higher prevalence of hearing loss (*P* = 0.001; beta: −0.10; B: −1.38; 95% CI: −2.20, −0.57).

**TABLE 2:** Associations (multivariable analysis) between the Mini-Mental State Examination (MMSE) score and other parameters in the Ural Very Old Study.

	Standardized regression coefficient beta	Non-standardized regression coefficient B	95% confidence interval of B	*P*-value	Variance inflation factor
Age (years)	−0.13	−0.29	−0.42, −0.16	<0.001	1.05
Region of habitation (rural/urban)	0.15	2.19	1.28, 3.09	<0.001	1.34
Level of education (illiteracy (no reading ability at all)/passing of the 5th class/passing of the 8th class/passing of the 10th class/passing of the 11th class/graduation/post-graduation	0.19	0.58	0.39, 0.77	<0.001	1.27
Depression score	−0.33	−0.21	−0.25, −0.17	<0.001	1.11
Visual acuity [logarithm of the minimal angle of resolution (LogMAR)]	−0.10	−0.86	−1.37, −0.36	0.001	1.13
In your leisure time, do you do any physically vigorous activities like running, strenuous sports or weight lifting for at least 10 mins at a time?	0.06	1.37	0.08, 2.65	0.04	1.03
Systolic blood pressure (mmHg)	0.13	0.03	0.02, 0.05	<0.001	1.53
Diastolic blood pressure (mmHg)	−0.08	−0.04	−0.07, −0.003	0.03	1.56

The statistical analysis consisted of a linear multivariable regression analysis with the MMSE score as the dependent parameter and as independent variables, all parameters that were significantly (P < 0.05) correlated with the dependent variable in the univariate analyses (please see Supplementary Table 1 for detailed information).

In a multinomial logistic model, a higher stage of CI (mild, moderate, and severe stage) was associated with older age (*P* = 0.001), rural region of habitation (*P* < 0.001), higher level of education (*P* = 0.001), higher level of hearing loss (*P* = 0.01), higher prevalence of depression (*P* < 0.001) and blindness (*P* = 0.005), and lower prevalence of vigorous physical activity in the leisure time (*P* = 0.007). A higher prevalence of severe cognitive impairment (MMSE score ≤ 9 points; mean: 6.8%; 95% CI: 5.5, 8.1) was associated (multivariable analysis) with a lower level of education (*P* = 0.007), a higher depression score (*P* < 0.001), worse visual acuity (*P* = 0.009), and a higher hearing loss score (*P* = 0.004; Table 3). In that model, the parameters of region of habitation (*P* = 0.18), age (*P* = 0.12), sex (*P* = 0.99), and diabetes (*P* = 0.63) were not significantly related with the prevalence of severe cognitive impairment. A higher prevalence of moderate cognitive impairment (MMSE score 10–18 points; 17.1%; 95% CI: 15.1, 19.0) (after exclusion of participants with severe cognitive impairment) was associated with younger age (OR: 1.18; 95% CI: 1.12, 1.24; *P* < 0.001), rural region of habitation (OR: 1.88; 95% CI: 1.29, 2.73; *P* = 0.001), lower level of education (OR: 0.81; 95% CI: 0.74, 0.88; *P* < 0.001), and higher depression score (OR: 1.08; 95% CI: 1.06, 1.10; *P* < 0.001). In that model, the parameters of sex (*P* = 0.29), diabetes (*P* = 0.61), visual acuity (*P* = 0.22) and hearing loss score (*P* = 0.11) were not significantly related with the prevalence of moderate cognitive impairment. In a similar manner, the prevalence of any CI (defined as a MMSE < 24) was associated with older age (OR: 1.07; 95% CI: 1.02, 1.13; *P* = 0.004), rural region of habitation (OR: 1.57; 95% CI: 1.12, 2.19; *P* = 0.009), lower level of education (OR: 0.78; 95% CI: 0.73, 0.84; *P* < 0.001), and worse best corrected visual acuity (OR: 1.39; 95% CI: 1.09, 1.76; *P* = 0.007).

**TABLE 3:** Associations (multivariable analysis) between the prevalence of severe cognitive impairment (defined as a Mini-Mental State Examination score of ≤ 9 points) (found in 98/1,442 (6.8%) participants and other parameters in the Ural Very Old Study).

	Odds ratio	95% confidence interval	*P*-value
Level of education (illiteracy (no reading ability at all)/passing of the 5th class/passing of the 8th class/passing of the 10th class/passing of the 11th class/graduation/post-graduation	0.79	0.66, 0.94	0.007
Depression score	1.10	1.07, 1.13	<0.001
Visual acuity (logMAR)	1.51	1.11, 2.07	0.009
Hearing loss score	1.03	1.01, 1.05	0.004

The statistical analysis consisted of a binary multivariable regression analysis with the prevalence of severe cognitive impairment as the dependent variable and as independent variables, all parameters that were significantly (P < 0.05) correlated with the dependent variable in the univariate analyses (please see Supplementary Table 1 for detailed information).

## Discussion

In our population-based study on very elderly individuals, the prevalence of any CI was 48.6%, with the mild, moderate and severe stage of CI having a prevalence of 24.8, 17.1, and 6.8%, respectively. A lower MMSE score was associated with older age, rural region of habitation, lower level of education, higher depression score, worse visual acuity, lower physical activity, lower systolic and higher diastolic blood pressure. If the parameter of depression was dropped, a lower MMSE was additionally associated with a higher prevalence of hearing loss. Correspondingly, a higher prevalence of severe CI was associated with a lower educational level, higher depression score, worse visual acuity, and higher hearing loss score.

The factors associated with a higher prevalence of CI in our study population were also found to be correlated with the CI prevalence in other study samples (Li et al., 2014; Coyle et al., 2017; Turusheva et al., 2017; Wu et al., 2017; Dominguez et al., 2018; Frank et al., 2019; Livingston et al., 2020; Huang et al., 2021; Shukla et al., 2021). Out of the list of modifiable risk factors mentioned by the 2020 Lancet commission on dementia prevention, the parameters of low education, hypertension, hearing impairment, depression, and physical inactivity were associated with a higher CI prevalence in our study (Livingston et al., 2020). In contrast, the factors of smoking, obesity, diabetes, social isolation and excessive alcohol consumption, which were also listed by the Lancet commission, were not associated with the CI prevalence in our investigation. A major reason for the discrepancy between our study and the Lancet commission’s list may be the design of our study as a cross-sectional investigation, which could not assess causal relationships in a longitudinal manner. In particular, risk factors for CI might have led to an early death, so that a survival bias might have prevented factors such as diabetes, obesity, smoking and excessive alcohol consumption from being associated with a higher prevalence of CI in our very old study population. In a similar manner, another potentially major reason for the discrepancy between the results presented by the Lancet commission and the findings obtained in our investigation may be the relatively high minimal age of 85 years of our study population, while most previous population-based studies had a minimal age of 50 or 60 years as inclusion criterion. It may also be the reason why the CI prevalence in our study population was not related with older age, since all study participants were very old and the range of age within the study population was relatively small. The finding may suggest that beyond an age of 85 years, the number of individuals dying with CI is similar to the number of individuals with incident CI. The reason why living alone (as was the case for 523 participants of our study) was not significantly related with CI in our study may have been that the family ties are usually strong in the society of Bashkortostan, so that the children and grandchildren take care of their elderly family members, in particular when these are living alone. Living alone may thus not mean social isolation which has been listed as a risk factor for CI by the 2020 Lancet commission on dementia prevention.

Of interest is the association between a lower MMSE score (and higher CI prevalence) with lower vision and lower hearing capacity. It agrees with observations made by Kuo et al. (2021) who reported on an 1. 9-, 1. 1-, and 2.0-fold increase in the cross-sectional hazard of dementia for self-reported functional vision impairment, hearing impairment, and dual sensory impairment in US adults, respectively. It is also in agreement with the results of the Health and Retirement Study, a longitudinal study of older US adults, that self-reported visual impairment, hearing impairment, and dual sensory impairment were associated with higher hazards of incident dementia (Maharani et al., 2020). It also conforms with the findings made in the English Longitudinal Study of Aging, in which individuals with poor and moderate self-reported hearing had a 57 and 39% higher hazard of incident dementia during a follow-up of 9 years, respectively (Davies et al., 2017). The findings also agree with observations made in the Beijing Eye Study, in which higher cognitive function was associated with a lower amount of undercorrection of refractive error (Xu et al., 2014). Several reasons for the association of CI and sensory impairment may be named, such as a sensory impairment-related reduction in external stimuli for cognitive activities; an increased risk of social isolation, depression, and reduced physical activity, with all these parameters being risk factors for the development of CI; a common mechanism like microvascular insufficiency for sensory impairment and CI; and the possibility of a sensory impairment as a consequence of CI in patients with CI and cataract as cause for vision impairment and who do not have the means, support or willingness for cataract surgery to be performed (Li et al., 2014; Coyle et al., 2017; Dominguez et al., 2018; Frank et al., 2019; Livingston et al., 2020; Huang et al., 2021; Shukla et al., 2021). Since our study was a cross-sectional investigation, it did not allow drawing conclusions on a causality between associated parameters. The findings of our study support, however, observations made in longitudinal investigations such as in the Aging and Cognitive Health Evaluation in Elders trial, in which treatment of hearing impairment was associated with a slowing of memory decline (Deal et al., 2017). In a similar manner, a longitudinal observation found that poor vision was associated with the development of dementia (*P* = 0.005), while individuals with very good or excellent vision at baseline had a 63% reduced risk of dementia over a mean follow-up period of 8.5 years (Rogers and Langa, 2010). A higher MMSE correlated with urban versus rural region of habitation in the multivariable analysis (Table 2). A major cause for this relationship may have been differences in the quality of the health care system between the rural region and the urban region, with a markedly less developed health care system in the rural region.

The prevalence of any, mild, moderate and severe CI as observed in our study cannot fully be compared with the results of previous investigations since most of them had study populations considerably younger than ours (Gbd, 2016a,^2019^; Wu et al., 2017; Dominguez et al., 2018; Gbd 2017 Us Neurological Disorders Collaborators et al., 2021). In addition, the definition of CI and dementia varied between the investigations. In the (World Alzheimer Report, 2015), the prevalence of dementia in the age group of 85+ years ranged between 24.7% in Central Asia and 30.7% in the Caribbean (World Alzheimer Report, 2015). In the study conducted by Skoog et al. (2017), the prevalence of dementia was 29.8% in 1986–1987 and 21.7% in 2008–2010. Taking account of the various stages of CI, these figures correspond to the prevalence of any, mild, moderate and severe CI in our study population of 48.6% (95% CI: 46.0, 51.2), 24.8% (95% CI: 22.5, 27.0), 17.1% (95% CI: 15.1, 19.0), and 6.8% (95% CI: 5.5, 8.1), respectively.

When the results of our study are discussed, its limitations should be taken into account. First, although 1,526 out of 1,882 eligible individuals participated in the Ural Very Old Study, resulting in a participation rate of 81%, the present investigation included only 1,442 (94.5%) individuals who underwent the MMSE. If one considers, however, the relatively old age of our study population with a minimum age of 85 years and the multimorbidity often occurring in that age, the participation rate for the clinical examinations in our study population may have been acceptable. Second, we did not collect information about the non-participating, yet eligible individuals, so that we could not assess differences between the participating individuals and the non-participating subjects. It prevented detecting a confounding factor due a potential bias in the inclusion of participants. Third, the cross-sectional design of our investigation allowed to draw conclusions only on associated factors, but not on causative factors. It may also have led to a survival bias with a preferential drop-out of individuals with CI caused by factors which by themselves were associated with increased mortality. Fourth, the study regions of our study were characteristic for Southern Russia with respect to demography, culture and lifestyle, geography, and climate. The percentage of Russians was lower in our study population than in populations from North-Western Russia and Central Russia. In the multivariable analysis, however, the ethnic background was not associated with the prevalence of CI, so that the relatively high percentage of non-Russians in the total study population might not have markedly influenced the results. Fifth, we did not differentiate between the various forms of CI and dementia, such as Alzheimer’s disease, Parkinson’s disease dementia, alcohol-related dementia or vascular-induced dementia. Sixth, the definition of the various degrees of CI by using the cut-off values of 19–23 points, 10–18 points, and ≤9 points were not adjusted for the dependencies of the MMSE score on parameters such as age and sex. Seventh, participants with a moderate to severe degree of CI might have had difficulties to fully understand the questions of the interview. They were helped, however, by family members or study personal who took extra time for the examination of these study participants. Eighth, we assessed associations between the MMSE and a multitude of various parameters without primarily following a hypothesis, but with the goal to explore associations of CI with medical, socioeconomic and other parameters. Since such an endeavor carries the risk of spurious results, we assessed interdependencies between the variables in the multivariable analysis, and additionally carried out a Bonferroni correction to adjust for performing multiple statistical analyses. Ninth, we assessed hearing loss only by the questionnaire, but not by audiometry. Tenth, we defined the various stages of CI based on cut-off values published in previous studies and did not conduct a validation study specifically for our study population (Crum et al., 1993; Creavin et al., 2016; Arevalo-Rodriguez et al., 2021).

Strengths of our investigation are that it is one of few population-based investigations on CI prevalence in a very old population; that also the inhabitants of all retirement homes in the study regions were included into the study; that a multitude of systemic parameters was assessed and included into the multivariable statistical analysis; and that the study was conducted in an ethnic and socio-economically diverse population.

In conclusion, in this elderly, multi-ethnic study population from rural and urban Russia, the prevalence of any, mild, moderate and severe CI was 48.6, 24.8, 17.1, and 6.8%, respectively. Besides internal medical and lifestyle factors, vision and hearing impairment were major factors associated with CI. Measures improving sensory impairment, in addition to addressing other risk factors, are warranted in elderly populations.

## Data availability statement

The raw data supporting the conclusions of this article will be made available by the authors, without undue reservation.

## Ethics statement

The studies involving human participants were reviewed and approved by the Ethics Committee of the Academic Council of the Ufa Eye Research Institute approved the study and informed written consent was obtained from all participants. The patients/participants provided their written informed consent to participate in this study.

## Author contributions

MB, GK, and JJ: design. MB: funding. SP-J and JJ: statistical analysis and first draft of the manuscript. MB, GK, EI, SP-J, AF, AT, IR, and JJ: data assessment, revision, and final approval of the submitted version.

## Conflict of interest

The authors declare that the research was conducted in the absence of any commercial or financial relationships that could be construed as a potential conflict of interest.

## Publisher’s note

All claims expressed in this article are solely those of the authors and do not necessarily represent those of their affiliated organizations, or those of the publisher, the editors and the reviewers. Any product that may be evaluated in this article, or claim that may be made by its manufacturer, is not guaranteed or endorsed by the publisher.
