# Supplementary material for: Cognitive impairment in the population-based ural very old study
Source: Front Aging Neurosci. 2022 Jul 19;14:912755. doi: 10.3389/fnagi.2022.912755 (PMC9344888; doi:10.3389/fnagi.2022.912755)
Supplement: Supplementary file 1 [file Table_1.docx]

**Supplemental Material**

Supplemental Table 1

Associations (univariable analysis) between the Mini Mental State Examination score and other parameters in the Ural Very Old Study

|  | Standardized Regression Coefficient beta | Non-Standardized Regression Coefficient B | 95% Confidence Interval of B | *P*-Value |
| --- | --- | --- | --- | --- |
| Age (years) | -0.19 | -0.45 | -0.57, -0.33 | <0.001 |
| Gender (men/women) | -0.06 | -0.98 | -1.78, -0.18 | 0.02 |
| Region of habitation (rural / urban) | 0.17 | 2.89 | 2.00, 3.78 | <0.001 |
| Ethnicity (non-Russian / Russian) | 0.06 | 0.76 | 0.04, 1.48 | 0.04 |
| Body height (cm) | -0.03 | -0.02 | -0.07, 0.02 | 0.29 |
| Body weight (kg) | 0.08 | 0.04 | 0.01, 0.08 | 0.02 |
| Body mass index (kg/m^2^) | 0.11 | 0.15 | 0.06, 0.24 | 0.001 |
| Waist circumference (cm) | 0.13 | 0.07 | 0.04, 010 | <0.001 |
| Hip circumference (cm) | 0.10 | 0.06 | 0.02, 0.09 | 0.002 |
| Waist/hip circumference ratio | 0.07 | 5.21 | 0.56, 9.86 | 0.03 |
| Level of education | 0.27 | 0.87 | 0.71, 1.03 | <0.001 |
| Smoking, currently | -0.01 | -0.91 | -4.93, 3.11 | 0.66 |
| Alcohol consumption, any | 0.07 | 1.40 | 0.31, 2.50 | 0.01 |
| Number of daily meals | 0.09 | 0.68 | 0.27, 1.10 | 0.001 |
| In a week how many days do you eat fruits? | 0.20 | 0.69 | 0.52, 0.86 | <0.001 |
| In a week how many days do you eat vegetables? | 0.04 | 0.16 | -0.08, 0.39 | 0.19 |
| Type of oil for cooking used: vegetable cooking oil – animal fat (butter) | -0.12 | -1.93 | -2.78, -1.08 | <0.001 |
| Food containing whole grains (no / yes) | 0.03 | 0.80 | -0.67, 2.27 | 0.29 |
| Salt consumed per day (g) | 0.10 | 0.32 | 0.14, 0.50 | 0.001 |
| Degree of processing meat (weak – medium – strong) | -0.19 | -2.23 | -2.83, -1.64 | <0.001 |
| Number of cups of coffee taken daily | 0.05 | 0.30 | -0.29, 0.90 | 0.31 |
| Number of cups of tea taken daily | 0.01 | 0.07 | -0.24, 0.37 | 0.67 |
| Preference of green or black tea | -0.12 | -3.60 | -5.31, -1.89 | <0.001 |
| In your leisure time, do you do any physically vigorous activities like running, strenuous sports or weight lifting for at least 10 minutes at a time? | 0.14 | 3.33 | 1.95, 4.71 | <0.001 |
| In your leisure time, do you do any moderate intensity activities like brisk walking, cycling or swimming for at least 10 minutes at a time? | 0.16 | 2.17 | 1.47, 2.86 | <0.001 |
| Over the past 7 days, how much time did you spend sitting or reclining on a typical day? | -0.23 | -0.001 | -0.001, -0.001 | <0.001 |
| Self-reported history of angina pectoris | 0.04 | 1.19 | -0.36, 2.74 | 0.13 |
| Self-reported history of asthma | 0.001 | 0.03 | -2.46, 2.51 | 0.98 |
| Self-reported history of arterial hypertension | 0.14 | 2.27 | 1.46, 3.07 | <0.001 |
| Self-reported history of arthritis | -0.11 | -1.51 | -2.22, -0.79 | <0.001 |
| History of previous bone fractures | 0.05 | 0.71 | -0.02, 1.43 | 0.06 |
| Self-reported history of low back pain | 0.02 | 0.23 | -0.48, 0.94 | 0.53 |
| Self-reported history of thoracic spine pain | -0.03 | -0.42 | -1.18, 0.35 | 0.29 |
| Self-reported history of neck pain | -0.03 | -0.50 | -1.37, 0.37 | 0.26 |
| Self-reported history of headache | -0.09 | -1.15 | -1.85, -0.45 | 0.001 |
| Self-reported history of cancer | 0.04 | 1.03 | -0.27, 2.33 | 0.12 |
| Self-reported history of cardiovascular disorders including stroke | 0.08 | 1.09 | 0.37, 1.82 | 0.003 |
| Self-reported history of dementia | -0.43 | -11.0 | -12.2, 9.82 | <0.001 |
| Self-reported history of diabetes mellitus | 0.04 | 0.89 | -0.28, 2.06 | 0.14 |
| Self-reported history of diarrhea | 0.01 | 0.56 | 2.89, 4.00 | 0.75 |
| Self-reported history of iron-deficiency anemia | -0.05 | -1.71 | -3.33, -0.09 | 0.04 |
| Self-reported history of low blood pressure and hospital admittance | -0.06 | -3.40 | -6.18, 0.62 | 0.02 |
| Self-reported history of osteoarthritis | 0.09 | 1.53 | 0.62, 2.44 | 0.001 |
| Self-reported history of skin disease | 0.01 | 0.28 | -1.19, 1.74 | 0.71 |
| Self-reported history of thyroid disease | 0.044 | 0.99 | -0.49, 2.47 | 0.19 |
| Self-reported history of falls | -0.004 | -0.06 | 0.76, 0.64 | 0.87 |
| Self-reported history of unconsciousness | -0.04 | -0.81 | -1.95, 0.33 | 0.17 |
| Age of the last menstrual bleeding (years) | 0.03 | 0.06 | -0.05, 0.17 | 0.27 |
| Age of last regular menstrual bleeding (years) | 0.06 | 0.11 | -0.002, 0.22 | 0.053 |
| Alanine aminotransferase (IU/L) | -0.01 | -0.01 | -0.05, 0.04 | 0.74 |
| Aspartate aminotransferase (IU/L) | -0.14 | -0.09 | -0.13, -0.05 | <0.001 |
| Aspartate aminotransferase / alanine aminotransferase ratio | -0.02 | -0.06 | -0.31, 0.19 | 0.63 |
| Bilirubin, total (µmol/L) | 0.04 | 0.03 | -0.02, 0.07 | 0.25 |
| High-density lipoproteins (mmol/L) | 0.10 | 0.83 | 0.35, 1.32 | 0.001 |
| Low-density lipoproteins (mmol/L) | -0.03 | -0.19 | -0.55, 0.17 | 0.31 |
| Cholesterol (mmol/L) | 0.09 | 0.42 | 0.12, 0.72 | 0.006 |
| Triglycerides (mmol/L) | 0.08 | 0.67 | 0.15, 1.18 | 0.01 |
| Rheumatoid factor (IU/mL) | 0.01 | 0.02 | -0.20, 0.24 | 0.85 |
| Erythrocyte sedimentation rate (mm / hour) | -0.05 | -0.02 | -0.06, 0.01 | 0.14 |
| Glucose (mmol/L) | -0.07 | -0.24 | -0.47, -0.02 | 0.03 |
| Creatinine (µmol/L) | 0.07 | 0.02 | 0.002, 0.04 | 0.03 |
| Urea (mmol/L) | 0.06 | 0.16 | -0.01, 0.33 | 0.07 |
| Residual nitrogen (g/L) | 0.05 | 4.65 | -0.86, 10.2 | 0.10 |
| Total protein (g/L) | 0.03 | 0.02 | -0.03, 0.07 | 0.41 |
| International normalized ratio (INR) | -0.01 | -0.26 | -3.25, 2.74 | 0.87 |
| Prothrombin time (%) | 0.02 | 0.01 | -0.03., 0.05 | 0.59 |
| Hemoglobin | 0.03 | 0.01 | -0.01, 0.03 | 0.27 |
| Erythrocytes (10^6^ cells / µL) | 0.03 | 0.37 | -0.37, 1.12 | 0.32 |
| Leukocytes (10^9^ cells / L) | 0.08 | 0.31 | 0.07, 0.55 | 0.01 |
| Rod-core granulocyte (% of leukocytes) | -0.02 | -0.08 | -0.32, 0.16 | 0.50 |
| Segment nuclear granulocyte (% of leukocytes) | 0.002 | 0.002 | -0.05, 0.06 | 0.96 |
| Eosinophil granulocytes (% of leukocytes) | -0.05 | -0.26 | -0.65, 0.13 | 0.19 |
| Lymphocytes (% of leukocytes) | 0.03 | 0.03 | -0.03, 0.09 | 0.37 |
| Monocytes (% of leukocytes) | 0.002 | 0.006 | -0.15, 0.16 | 0.94 |
| Prevalence of diabetes mellitus | -0.04 | -0.62 | -1.61, 0.38 | 0.23 |
| Anemia (serum hemoglobin concentration <140 g/L in men, <130 g/L in women) | -0.01 | -0.11 | -0.88, 0.67 | 0.79 |
| Blood pressure, systolic | 0.06 | 0.02 | 0.001, 0.03 | 0.04 |
| Blood pressure, diastolic | -0.07 | -0.03 | -0.06, -0.01 | 0.01 |
| Blood pressure, mean | -0.01 | -0.004 | -0.03, 0.02 | 0.74 |
| Arterial hypertension | 0.08 | 1.50 | 0.40, 2.60 | 0.008 |
| Arterial hypertension, stages | 0.07 | 0.45 | 0.08, 0.81 | 0.02 |
| Self-reported history of prevalence of chronic obstructive pulmonary disease | 0.02 | 0.55 | -0.77, 1.87 | 0.41 |
| Ankle-brachial index, right | -0.04 | -3.92 | -10.0, 2.19 | 0.21 |
| Ankle-brachial, left | -0.06 | -5.41 | -11.4, 0.57 | 0.08 |
| Metabolic syndrome | 0.02 | 0.20 | -0.58, 0.97 | 0.62 |
| Hearing loss score | -0.13 | -0.06 | -0.08, -0.03 | <0.001 |
| Depression Score | -0.42 | -0.27 | -0.30, -0.24 | <0.001 |
| State-Trait Anxiety Inventory | -0.34 | -0.21 | -0.24, -0.18 | <0.001 |
| Manual dynamometry, right hand | 0.26 | 0.22 | 0.17, 0.27 | <0.001 |
| Manual dynamometry, left hand | 0.25 | 0.23 | 0.17, 0.28 | <0.001 |
| Refractive error, spherical equivalent (diopters) | -0.003 | -0.005 | -0.14, 0.13 | 0.94 |
| Best corrected visual acuity (logarithm of the minimal angle of resolution (LogMAR)) | -0.23 | -2.13 | -2.66, -1.59 | <0.001 |
